# X-ray structure of the *Rhodobacter sphaeroides* reaction center with an M197 Phe→His substitution clarifies the properties of the mutant complex

**DOI:** 10.1107/S2052252521013178

**Published:** 2022-02-01

**Authors:** Georgii Selikhanov, Tatiana Fufina, Sebastian Guenther, Alke Meents, Azat Gabdulkhakov, Lyudmila Vasilieva

**Affiliations:** a Group of Structural Studies of Macromolecular Complexes, Institute of Protein Research, Russian Academy of Sciences, Institutskaya 4, Pushchino 142290, Moscow Region, Russian Federation; b Federal Research Center Pushchino Scientific Center for Biological Research PSCBR, Institute of Basic Biological Problems, Russian Academy of Sciences, Institutskaya 2, Pushchino 142290, Moscow Region, Russian Federation; c Center for Free-Electron Laser Science CFEL, Deutsches Elektronen-Synchrotron DESY, Notkestrasse 85, 22607 Hamburg, Germany; d Deutsches Elektronen-Synchrotron DESY, Notkestrasse 85, 22607 Hamburg, Germany

**Keywords:** photosynthetic reaction center, *Rhodobacter sphaeroides*, X-ray serial crystallography, hydrogen-bonding networks, redox potential, protein structure, membrane proteins

## Abstract

The first steps of the global process of photosynthesis take place in specialized membrane pigment–protein complexes called photosynthetic reaction centers (RCs). The RC of the photosynthetic purple bacterium *Rhodobacter sphaeroides*, a relatively simple analog of the more complexly organized photosystem II in plants, algae and cyanobacteria, serves as a convenient model for studying pigment–protein interactions that affect photochemical processes. In bacterial RCs the bacteriochlorophyll (BChl) dimer P serves as the primary electron donor, and its redox potential is a critical factor in the efficient functioning of the RC. It has previously been shown that the replacement of Phe M197 by His strongly affects the oxidation potential of P (*E*_m_ P/P^+^), increasing its value by 125 mV, as well as increasing the thermal stability of RC and its stability in response to external pressure. The crystal structures of F(M197)H RC at high resolution obtained using various techniques presented in this report clarify the optical and electrochemical properties of the primary electron donor and the increased resistance of the mutant complex to denaturation. The electron-density maps are consistent with the donation of a hydrogen bond from the imidazole group of His M197 to the C2-acetyl carbonyl group of BChl P_B_. The formation of this hydrogen bond leads to a considerable out-of-plane rotation of the acetyl carbonyl group and results in a 1.2 Å shift of the O atom of this group relative to the wild-type structure. Besides, the distance between BChl P_A_ and P_B_ in the area of pyrrole ring I was found to be increased by up to 0.17 Å. These structural changes are discussed in association with the spectral properties of BChl dimer P. The electron-density maps strongly suggest that the imidazole group of His M197 accepts another hydrogen bond from the nearest water molecule, which in turn appears to form two more hydrogen bonds to Asn M195 and Asp L155. As a result of the F(M197)H mutation, BChl P_B_ finds itself connected to the extensive hydrogen-bonding network that pre-existed in wild-type RC. Dissimilarities in the two hydrogen-bonding networks near the M197 and L168 sites may account for the different changes of the *E*_m_ P/P^+^ in F(M197)H and H(L168)F RCs. The involvement of His M197 in the hydrogen-bonding network also appears to be related to stabilization of the F(M197)H RC structure. Analysis of the experimental data presented here and of the data available in the literature points to the fact that the hydrogen-bonding networks in the vicinity of BChl dimer P may play an important role in fine-tuning the redox properties of the primary electron donor.

## Abstract 2

Crystal structures of an F(M197)H mutant form of the photosynthetic reaction center obtained using different techniques clarify the optical and electrochemical properties of the primary electron donor and the increased resistance of this mutant complex to denaturation.

## 1. Introduction

**Figure 1:**
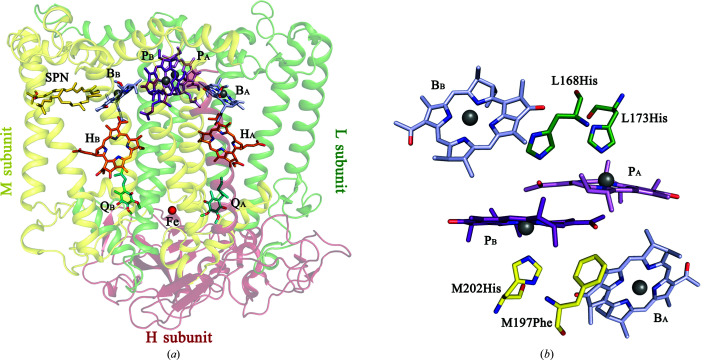
(*a*) Overall structure of *R. sphaeroides* RC (PDB entry 3v3y). P_A_ and P_B_, special pair of bacteriochlorophylls; B_A_ and B_B_, monomeric bacteriochlorophylls; H_A_ and H_B_, bacteriopheophytins; Q_A_ and Q_B_, quinones; SPN, carotenoid spheroidene. (*b*) Bacteriochlorophylls in the structure of wild-type *R. sphaeroides* RC. The view is along the axis of twofold symmetry from the periplasmic side of the membrane. His L168 and Phe M197 near the acetyl carbonyl groups of BChls P_A_ and P_B_ are shown. His L173 and His M202 are ligands of BChls P_A_ and P_B_, respectively.

During the process of photosynthesis, light energy is converted into the energy of chemical bonds. The first steps in this global process take place in specialized membrane pigment–protein complexes known as photosynthetic reaction centers (RCs). The RC from the purple bacterium *Rhodobacter sphaeroides* is an integral membrane pigment–protein complex that consists of three subunits and ten cofactors. The cofactors are arranged in two membrane-spanning branches, A and B, around an axis of pseudo-twofold symmetry. Only one, the A branch, is active in photosynthetic electron transfer. The RC includes two bacteriochlorophylls (BChls), P_A_ and P_B_, combined into a special pair P, two monomeric BChls B_A_ and B_B_, two bacteriopheophytins (BPhe) H_A_ and H_B_, two quinones and a nonheme Fe atom [Allen *et al.*, 1987 ▸; Fig. 1 ▸(*a*)]. The cofactors interact with the surrounding amino-acid residues through relatively weak hydrophobic, electrostatic, van der Waals contacts. Through these interactions, the RC protein participates in fine-tuning the biophysical properties of the cofactors (for a review, see Leonova *et al.*, 2011 ▸).

Being the primary electron donor, the BChl dimer P is a crucial component in the RC, and the redox potential of P is a critical factor in the efficient functioning of the RC. It has been shown that in *R. sphaeroides* RC the value of *E*_m_ P/P^+^ can be altered in the range +410 to +765 mV by changing the hydrogen-bond interactions of the P carbonyl groups with their protein environment (for a review, see Allen & Williams, 1995 ▸). These are the two C2-acetyl carbonyl groups of rings I and the two C9-keto groups of rings V conjugated to the π electronic system of the P_A_ and P_B_ macrocycles. Extensive studies of so-called hydrogen-bond mutants with amino-acid substitutions near the carbonyl groups has accumulated a great deal of data, but some questions remain to be answered. The strongest effects on the *E*_m_ P/P^+^ were demonstrated by substitutions of His L168 and Phe M197, which are symmetry-related amino-acid residues near the acetyl carbonyl groups. In wild-type (WT) *R. sphaeroides* RC the only hydrogen bond that connects the special pair P to the surrounding protein is the hydrogen bond that His L168 donates to the C2-acetyl carbonyl group of BChl P_A_ [Fig. 1 ▸(*b*)]. The strength of this bond is 4.9 kcal mol^−1^ as estimated by FT-Raman spectroscopy (Mattioli *et al.*, 1994 ▸). Breakage of this hydrogen bond resulted in a 95 mV decrease in the *E*_m_ P/P^+^ (Lin *et al.*, 1994 ▸). Replacement of the symmetry-related Phe M197 with His introduced a new hydrogen bond to the acetyl carbonyl group of BChl P_B_ (3.5 kcal mol^−1^; Ridge *et al.*, 2000 ▸) and increased the *E*_m_ P/P^+^ by 125 mV, which is much greater than the decrease in the potential brought about by the H(L168)F mutation (Lin *et al.*, 1994 ▸; Mattioli *et al.*, 1994 ▸). To date, no data are available that can explain the dissimilar effects of His residues at these two symmetry-related positions on the *E*_m_ P/P^+^, but these effects imply that there might be other factors that participate in the tuning of the redox properties of the BChl dimer. Besides, considering the previously stated assumption about the possible out-of-plane rotation of the acetyl carbonyl group of BChl P_B_ as a result of hydrogen bonding to His M197 (Ivancich *et al.*, 1998 ▸), it is unclear why the energy of the P Q_*y*_ transition in the absorption spectrum of F(M197)H RC remained practically unchanged, while in other hydrogen-bond mutants with a proven rotation of the group and a less pronounced effect on the oxidation potential of P notable shifts of the P Q_*y*_ band were observed (Ridge *et al.*, 2000 ▸).

In recent work, the thermal stability and the stability in response to external pressure were compared for the WT *R. sphaeroides* RC and its H(L168)F and F(M197)H mutants (Holden-Dye *et al.*, 2011 ▸; Kangur *et al.*, 2017 ▸). It was suggested that the introduction of a hydrogen bond between His M197 and the acetyl carbonyl group of P_B_ made F(M197)H RC more resistant to both thermal denaturation and external pressure, while breaking the existing hydrogen bond made by His L168 and the acetyl carbonyl group of P_A_ had the opposite effect. Based on the structure of a quintuple mutant including the F(M197)H substitution with a resolution limit of 4.5 Å (Thielges *et al.*, 2005 ▸), the authors assumed that the pigment–protein interactions that arose as a result of the introduction of His at the M197 site could be similar to those of His L168 with its environment (Holden-Dye *et al.*, 2011 ▸). However, this assumption is not consistent with the differing effects of His L168 and His M197 on the redox properties of the BChl P dimer. To clarify this issue, a crystal structure of the mutant RC at high resolution is required.

In the present work, we report X-ray crystal structures of F(M197)H RC for the first time, which were obtained using various techniques at different temperatures at high resolution; on the basis of these structures, we discuss how the changes in the structure of the protein that are observed can account for the previously reported optical and electrochemical properties of the primary electron donor and the increased stability of F(M197)H RC.

## 2. Materials and methods

### 2.1. Site-directed mutagenesis, cell growth and purification of the reaction center

The F(M197)H mutation was introduced using the genetic system for site-directed mutagenesis, as described previously (Khmelnitskiy *et al.*, 2013 ▸). An altered *pufM* gene was shuttled into the broad-host-range vector, a derivative of pRK415, containing an EcoRI–HindIII DNA fragment that included the *pufBALMX* genes. The resulting plasmid was transformed into *R. sphaeroides* strain DD13 (Jones *et al.*, 1992 ▸) through conjugative crossing to give a recombinant strain with the RC-LH1 phenotype. The control strain (pseudo wild-type) was made similarly using the DD13 strain complemented by a pRK415 derivative containing a wild-type copy of the *puf* genes. The growth of recombinant bacterial strains in dark semi-aerobic conditions has been described elsewhere (Khatypov *et al.*, 2005 ▸). The cells were harvested and then broken by ultrasonication, and membranes for reaction-center purification were harvested by ultracentrifugation. Reaction centers were solubilized using lauryldimethylamine oxide (LDAO) and were then purified on a DE52 anion-exchange cellulose column, followed by passage through Fractogel EMD DEAE (S) columns (Merck), as described in detail previously (Fufina *et al.*, 2007 ▸). The purity of the reaction centers at each step was estimated by absorbance spectroscopy using the ratio of protein absorbance at 280 nm to bacteriochlorophyll absorbance at 802 nm (*A*_280_/*A*_802_; Okamura *et al.*, 1974 ▸). Reaction centers with an *A*_280_/*A*_802_ of below 1.4 were sufficiently pure for crystallization.

### 2.2. Crystallization

For crystallization of F(M197)H RC, we applied an *in meso* approach using lipid sponge phase (LSP). LSP was prepared in the same way as described in Wadsten *et al.* (2006 ▸). We used the hanging-drop vapor-diffusion method. Drops consisted of 1 µl LSP, 1 µl protein solution (25 mg ml^−1^) and 1 µl 1.65 *M* sodium tricitrate, and were equilibrated against a reservoir solution consisting of 2 *M* potassium phosphate pH 7.5. Incubation was carried out at 16°C. Tetragonal crystals belonging to space group *P*4_2_2_1_2 appeared within five days and grew to a maximum size of 60–70 µm. For crystallization in larger volumes for serial crystallography experiments we used LSP crystallization in plastic tips, as described previously by Selikhanov *et al.* (2020 ▸).

It has previously been shown that during *in meso* crystallization using monoacylglycerols as matrix lipids the spheroidene cofactor is missing from the resulting crystal structure of the *R. sphaeroides* RC (Selikhanov *et al.*, 2020 ▸). To prevent this, we used co-crystallization with spheroidene as described in Selikhanov *et al.* (2020 ▸).

### 2.3. Data collection and processing, model building and refinement

X-ray diffraction data sets were collected using various techniques on beamline P11 at PETRA III, DESY, Hamburg, Germany. The first data set was collected in ‘classical’ style using a single crystal cryocooled to 100 K. A room-temperature (RT) data set was obtained by multi-crystal fixed-target crystallography using a Roadrunner I-type silicon chip (Lieske *et al.*, 2019 ▸). Crystals were directly applied onto the chip. Drying of the samples during data collection was prevented by sealing the chips with a cover containing two windows of 500 nm Mylar foil. Data were collected in 20° wedges at predefined grid points. The first 10° of each data set were processed using *XDS* (Kabsch, 2010 ▸). The best data sets were selected based on ISa > 15, and were subsequently merged using the genetic algorithm implemented in *CODGAS* (Zander *et al.*, 2016 ▸). The final data set contained 28 partial data sets.

Molecular replacement was performed by *Phaser* (McCoy *et al.*, 2007 ▸) using the coordinates of wild-type RC obtained by LSP co-crystallization with carotenoid (PDB entry 6z1j; Selikhanov *et al.*, 2020 ▸). Data-collection and refinement statistics are given in Tables 1 ▸ and 2 ▸. The initial models were subjected to crystallographic refinement using *REFMAC*5 (Murshudov *et al.*, 2011 ▸). Manual rebuilding of the models was carried out using *Coot* (Emsley *et al.*, 2010 ▸). Figures were prepared using *PyMOL* (DeLano, 2002 ▸). The coordinates and structure factors have been deposited in the Protein Data Bank with PDB codes 7od5 and 7p17. The estimated minimal and maximal coordinate errors were calculated by *SFCHECK* (Vaguine *et al.*, 1999 ▸). The calculated DPI value was estimated using the *Online_DPI* web server (Kumar *et al.*, 2015 ▸).

## 3. Results and discussion

To explore the structural consequences of the F(M197)H mutation in detail, X-ray crystal structures of the mutant RC were obtained under two different conditions: (i) cryo-conditions using a single crystal and (ii) multi-crystal crystallography at room temperature. The corresponding structures were determined to resolutions of 2.1 and 2.04 Å, respectively. The structures obtained using the different techniques are in good agreement for the positions of the atoms of the main chain. The difference between the structure at room temperature and that at 100 K exceeds 0.3 Å. After refinement, the structural models of F(M197)H RC were compared with the structures of the WT RC (2.1 Å) obtained from one crystal at 100 K (PDB entry 6z1j) and by multi-crystal crystallo­graphy at room temperature at 2.14 Å resolution (S. Guenther *et al.*, in preparation) that were crystallized under the same conditions. Data-collection and refinement statistics are shown in Tables 1 ▸ and 2 ▸. Since Phe is bulkier than His, the Phe→His substitution created no steric problems, and good structural conservation was observed throughout the bulk of the protein–cofactor system. Changes in the structure of the mutant RC were mainly confined to the side chain of the M197 His residue, to the C2-acetyl group of BChl P_B_, a conserved water molecule and Asp L155. It is important to mention that there is a significant difference between the F(M197)H mutant structures obtained at 100 K and at room temperature. In both of these structures we observed a rotation of the acetyl group of BChl P_B_ relative to the plane of the macrocycle that differs from that seen in the WT structure, but in the case of the room-temperature mutant there was a more pronounced rotation compared with the cooled structure. For further comparison, the structures of the WT and mutant RCs obtained at room temperature were selected, since these conditions are closer to physiological.

### 3.1. Structural consequences of the formation of a new hydrogen bond in F(M197)H RC

**Figure 2:**
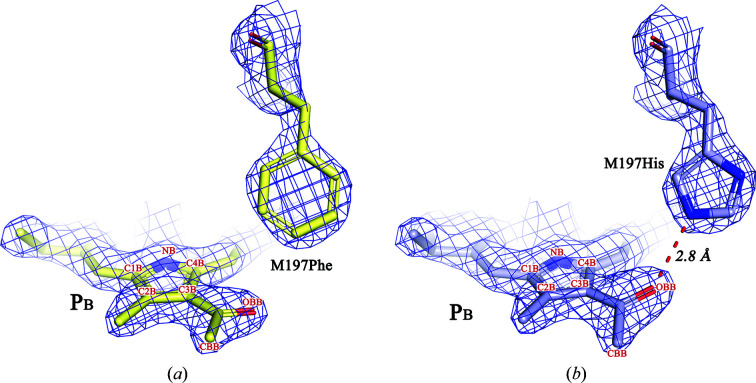
Electron-density maps for the WT RC (*a*) and F(M197)H RC (*b*) attributable to the M197 residue and the C2-acetyl group of BChl P_B_ and the fits of the structural models to the density. 2.1 Å resolution, 2.0σ [0.47 e Å^−3^ for the WT RC and 0.63 e Å^−3^ for F(M197)H RC].

**Figure 3:**
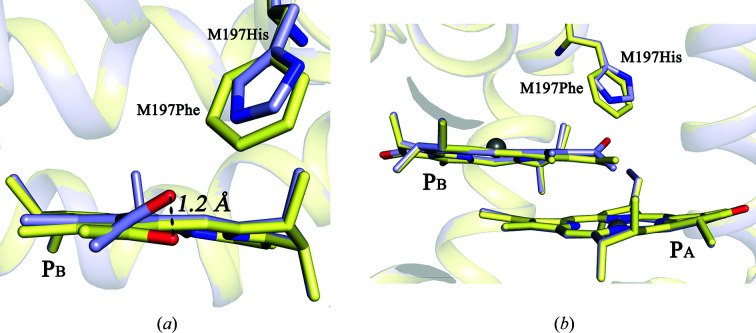
Superimposition of the C2-acetyl group of BChl P_B_ in the WT RC (pale yellow) and in F(M197)H RC (light blue). Views of this group are shown along the plane of BChl P_B_ (*a*) and perpendicular to the M197 imidazole ring (*b*).

Fig. 2 ▸ shows electron-density maps for the WT RC [Fig. 2 ▸(*a*)] and F(M197)H RC [Fig. 2 ▸(*b*)] attributable to the M197 residue and the C2-acetyl group of BChl P_B_, and the fits of the structural models to the density. The orientation of the introduced His M197 was similar to that of the native Phe, with the imidazole ring of the His residue directed towards the BChl P_B_ acetyl carbonyl group. The distance from the NE2 N atom of the His M197 imidazole group to the carbonyl O atom of the acetyl group was estimated to be 2.8 Å, which is consistent with the engagement of these groups in a hydrogen-bond interaction. Data favoring the formation of this hydrogen bond in F(M197)H RC have previously been obtained by FT-Raman spectroscopy (Mattioli *et al.*, 1994 ▸). The acetyl group of P_B_ in the WT RC structure from one crystal at 100 K sits slightly out of the plane of the BChl ring, showing a −7.4° out-of-plane clockwise rotation (not shown). In the F(M197)H RC structure from one crystal at 100 K, this group has undergone a significant rotation that places it approximately 20.7° out of plane, representing a 28.1° anti­clockwise rotation compared with that in the WT RC (or the displacement of the O atom by 1.2 Å; Fig. 3 ▸). Comparison of the structures obtained at room temperature shows a similar out-of-plane rotation of 33.4°, representing a total 32.8° anticlockwise rotation compared with that in the WT RC (0.6° out-of-plane anticlockwise rotation). The change in the orientation of this group in F(M197)H RC has previously been suggested in the work of Ivancich *et al.* (1998 ▸).

A 20° out-of-plane rotation of the acetyl group of P_B_ was observed in the structure of F(M197)R RC (Ridge *et al.*, 2000 ▸), and this was thought to weaken the degree of conjugation of this group with the π-electron system of the P BChls. The rotation of this group was assumed to explain the absence of a contribution of the acetyl carbonyl group of P_B_ to the FT-Raman spectrum of F(M197)R RC. Our data show that despite a much more pronounced out-of-plane rotation of the acetyl group in F(M197)H RC it remains conjugated to the P_B_ electron system, which is confirmed by the presence of this band in the corresponding FT-Raman spectrum (Ridge *et al.*, 2000 ▸). Quantum-mechanical calculations indicated that the Q_*y*_ absorption band of dimer P should be sensitive to the orientation of the C2-acetyl groups of the BChls with respect to the macrocycle planes (Parson & Warshel, 1987 ▸; Warshel & Parson, 1987 ▸; Thompson *et al.*, 1991 ▸). As has been discussed in these publications, an out-of-plane rotation of either of the two acetyl groups can cause a notable red shift of the P Q_*y*_ band. In disagreement with this prediction, out-of-plane rotation of the acetyl group in the structure of F(M197)R RC was accompanied by a 13–15 nm blue shift of the P Q_*y*_ band, while the similar out-of-plane rotation of this group in F(M197)H RC presented in this work has almost no effect on the position of the P Q_*y*_ band (as discussed below; Ridge *et al.*, 2000 ▸).

Previously, it was suggested that the absorbance properties of P could be sensitive to several other parameters, including very small changes in spacing between the two BChls of the dimer (Parson & Warshel, 1987 ▸; Thompson *et al.*, 1991 ▸). It is known that in structures of RCs from purple bacteria the two BChls forming P overlap in the ring I region, and the close distance of 3.5 Å results in them being electronically coupled (Allen *et al.*, 1987 ▸; Deisenhofer *et al.*, 1985 ▸). Examination of the mutant RC structure reveals that the distance between BChl P_A_ and P_B_ in the area of pyrrole ring I increases by approximately 0.10–0.17 Å depending on the pairs of atoms that are used for measurements (Table 3 ▸). Within the limits of the present resolution, with an error of 0.06–0.1 Å, the exact distance cannot be determined more accurately, but the data obtained allow the presence of such changes to be fixed in space between the two halves of the P dimer. Parson and Warshel calculated that even a small separation of the two halves of the P dimer would alter the energy of the P Q_*y*_ transition and predicted that an increase in the distance between P_A_ and P_B_ of 0.1 Å could result in a 15 nm blue shift of the P Q_*y*_ band (Parson & Warshel, 1987 ▸). Following this prediction, one might expect a similar shift in the absorption spectrum of F(M197)H RC, but only a 2 nm blue shift of the P Q_*y*_ band was detected (Ridge *et al.*, 2000 ▸). We assume that the energy of the P Q_*y*_ transition in the mutant RC could be affected both by the rotation of the C2-acetyl group of P_B_ and by a small increase in the distance between the two BChls of the dimer, and as a result of these opposing effects the position of the P Q_*y*_ band in the absorption spectrum of the mutant RC remained practically unchanged.

### 3.2. Hydrogen-bonding networks in the WT and F(M197)H RCs

**Figure 4:**
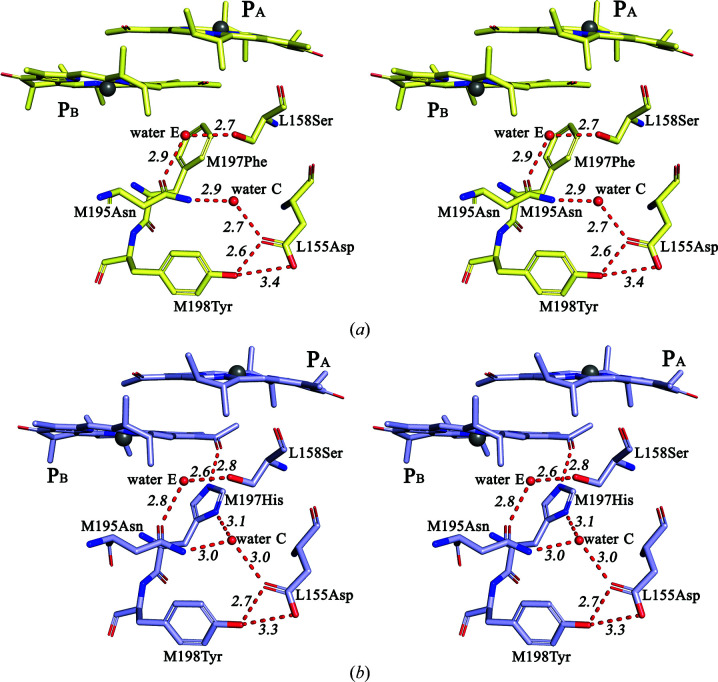
Stereoviews of the hydrogen-bonding networks in the vicinity of the M197 residue in the WT RC (*a*) and F(M197)H RC (*b*). All distances are shown in Å.

In the work of McAuley-Hecht and coworkers studying the structural perturbations caused by the F(M197)R mutation, it was noticed that in the structure of *R. sphaeroides* RC there is a cleft at the interface of the L and M subunits that extends from the periplasmic surface of the protein to the Phe M197 residue (McAuley-Hecht *et al.*, 1998 ▸). Residues Asn M195 and Tyr M198 from the M subunit and Asp L155, Leu L154, Val L157 and Ser L158 from the L subunit are located at the edges of this cleft; some of these residues are shown in Fig. 4 ▸(*a*). This cavity also accommodates two water molecules. One water that is close to the C9-keto-carbonyl group of BChl B_A_ was assigned as water A and was suggested to take part in photosynthetic electron transfer from P* to B_A_ (Potter *et al.*, 2005 ▸; Yakovlev *et al.*, 2005 ▸). Another water molecule makes hydrogen bonds to the amino group of Asn M195 and the carboxylic acid group of Asp L155 in the WT RC structure and in the structure of F(M197)H RC (Fig. 4 ▸) presented in this work. In the literature, there is no information available on the role of this conserved water in *R. sphaeroides* RC or other RCs from purple bacteria. For the sake of simplicity, in the following we will refer to this water as ‘water C’ by analogy with waters A and B near the C9-keto-carbonyl groups of monomeric BChls (Potter *et al.*, 2005 ▸; Jones, 2009 ▸). It is known that for a polar water molecule to be stably fixed inside of the hydrophobic membrane-protein interior it usually has to be involved in one or two hydrogen bonds to its surroundings (Jones, 2009 ▸). Examination of the WT RC structure close the periplasmic surface of the membrane revealed a widespread hydrogen-bonding network that involves residues of the L and M subunits in the protein environment of the P_B_ and B_A_ molecules [Fig. 4 ▸(*a*)]. This network includes Asp L155, Tyr M198, water C, Asn M195, a water molecule that will be assigned as water E and Ser L158. Fig. 4 ▸(*a*) shows the distances between the related side groups, which are all within the 2.5–3.4 Å range consistent with hydrogen-bond formation. The X-ray data presented in this work allow us to conclude on the establishment of such interactions with a high degree of probability.

**Figure 5:**
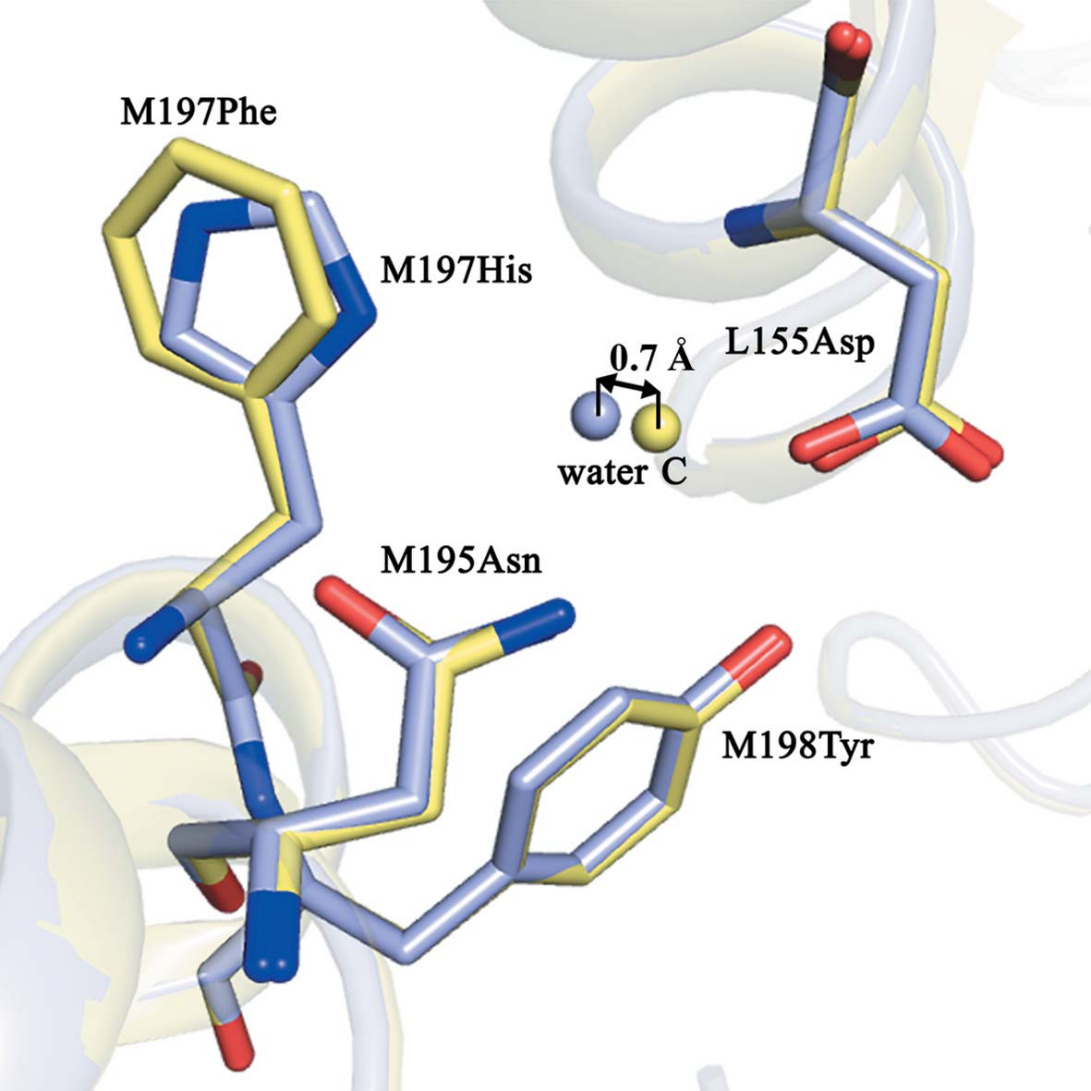
Superimposition of the structural models of the WT RC (pale yellow) and F(M197)H RC (light blue).

This hydrogen-bonding network appears to be undisturbed in the F(M197)H RC structure, with the distances between contacting side groups remaining within the range 2.5–3.2 Å [Fig. 4 ▸(*b*)]. Also, water C appears to donate a third hydrogen bond to the ND1 N atom of His M197, which is positioned 3.1 Å from the imidazole ring [Fig. 4 ▸(*b*)]. The notable shift of water C by some 0.7 Å towards the imidazole ring that is observed, together with a less pronounced shift of Asp L155 in a direction towards this water, supports the involvement of water C in a hydrogen-bond interaction with His M197 (Fig. 5 ▸). Thus, our results show that the Phe→His substitution connects the acetyl carbonyl group of BChl P_B_ to the existing hydrogen-bonding network through the imidazole group of His M197.

It is known that additional hydrogen-bonding networks can provide new electron-transfer pathways in proteins (Beratan *et al.*, 1991 ▸). Changes in the electron-conducting properties of a protein in the proximity of the primary electron donor and the nearest electron acceptor might be a possible reason for the unpredictably high rates of primary charge separation and quantum yields of this process reported for F(M197)R and F(M197)H mutant RCs, in contrast to the other known hydrogen-bond mutants, which demonstrated decreased rates of forward electron transfer (Ridge *et al.*, 2000 ▸; Wang *et al.*, 2007 ▸; Khmelnitskiy *et al.*, 2013 ▸).

### 3.3. Comparison of through-bond interactions near His M197 and His L168

**Figure 6:**
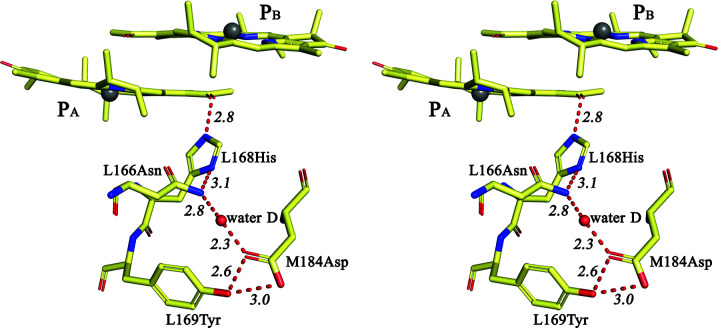
Stereoviews of the hydrogen-bonding network near His L168 in the *R. sphaeroides* RC structure. All distances are shown in Å.

In the WT RC structure, a similar but slightly more restricted cleft is present on the BChl P_A_ side near His L168. The conserved residues that line this cleft (Fig. 6 ▸) are symmetry-related to those in the M197 cleft, as shown in Fig. 4 ▸(*b*). The position of Asn L166 is related to Asn M195, that of Tyr L169 to Tyr M198, and that of Asp M184 to Asp L155. A conserved water, which is symmetry-related to water C and is assigned as ‘water D’, is present in this region of the protein. This water and its possible interaction with Asn L166 was previously mentioned in Ivancich *et al.* (1997 ▸). The protein environment of His L168 is similar to that of His M197, with a few important structural differences that should be noted. (i) The residue symmetry-related to Ser L158 is Asn M187, which makes no hydrogen-bond contacts with the L subunit. (ii) No water molecule symmetry-related to water E was found close to the carbonyl group of Asn L166. (iii) The side group of Asn L166 is much more extended into the cleft compared with that of Asn M195, displacing water D 5.6 Å away from the imidazole ring of His L168 (Fig. 6 ▸). As a result, water D does not interact with His L168, but all other contacts of this water with neighboring residues seem to be similar to those that water C makes with its surroundings. Previously, the inter­action of His L168 with Asn L166 was suggested by Ivancich *et al.* (1997 ▸). Subsequently, the formation of a hydrogen bond between the ND1 atom of His L168 and the amide group of Asn L166 was discussed by Holden-Dye *et al.* (2011 ▸). This issue will be discussed further below. Thus, Figs. 4 ▸(*b*) and 6 ▸ clearly show that the patterns of polar intermolecular interactions on the P_A_ and the P_B_ sides are similar but have some differences, and that there are two fewer interconnected hydrogen bonds near L168 His compared with those in the vicinity of M197 His.

### 3.4. Relationship between the structural changes and the oxidation potential of P in the F(M197)H RC

As outlined in Section 1, it has been noted in previous work that the formation of hydrogen bonds between the conjugated carbonyl groups of the P BChls and the surrounding protein has a significant influence on the oxidation potential of P (Allen & Williams, 1995 ▸). The combination of hydrogen bonds in double and triple mutants was shown to be additive (Lin *et al.*, 1994 ▸), and the increase of the P/P^+^ midpoint potential correlated with the total change in hydrogen-bonding energy (Mattioli *et al.*, 1995 ▸). In contrast, no simple correlation was found between the strength of the hydrogen bond and the *E*_m_ P/P^+^ in single hydrogen-bond mutants (Mattioli *et al.*, 1994 ▸). The substantial body of experimental data obtained on hydrogen-bond mutants has led researchers to the conclusion that modulation of the redox potential of P is a complex and multifactorial process that not only depends on hydrogen-bond formation (Ivancich *et al.*, 1998 ▸). In particular, for the residue pair M197 and L168, which most strongly affect the *E*_m_ P/P^+^, the oxidation potential of P was assumed to be correlated with the side-chain permanent dipoles of the residues present at these positions (Spiedel *et al.*, 2002 ▸). Surprisingly, this correlation was observed regardless of hydrogen-bond formation between the residues at these positions and the acetyl carbonyl groups. As discussed in Spiedel *et al.* (2002 ▸), the imidazole ring possesses a permanent dipole of 4.66 D, which is much higher than the permanent dipoles of other residues (Sandberg & Edholm, 2001 ▸), and this was thought to explain its strong effect on the oxidation potential of P. This approach, however, does not clarify why the opposite mutations F(M197)H and H(L168)F at two symmetry-related positions produce different changes in the *E*_m_ P/P^+^ of +125 and −95 mV, respectively (Lin *et al.*, 1994 ▸; Mattioli *et al.*, 1994 ▸). It is also unclear why the introduction of His at another pair of positions, L131 and M160, near the C9-keto-groups of P, which are also conjugated with the π-electron system of the BChl dimer, brought about smaller changes of the *E*_m_ P/P^+^ of +80 and +60 mV, respectively (Lin *et al.*, 1994 ▸; Mattioli *et al.*, 1994 ▸).

In the F(M197)R RC, the dipole moment of Arg M197 (2.39 D), although correlated with an increase in the oxidation potential of P (+78 mV), apparently did not affect it because the side group of this residue did not contact the acetyl group of BChl P_B_ in any way. In the previously reported structure of this mutant RC, the side group of Arg M197 was turned towards Asp L155 and was suggested to form a salt-bridge interaction with this residue (Ridge *et al.*, 2000 ▸). The Phe→Arg substitution at the M197 site altered its environment compared with the WT RC structure. A new water molecule was observed in the vicinity of the acetyl carbonyl group that was positioned close enough for the formation of a hydrogen bond. The dipole moment of a single water molecule (1.85 D) is similar to that of Tyr (1.83 D), while the changes in the *E*_m_ P/P^+^ in the F(M197)R and F(M197)Y RCs differed at +80 and +30 mV, respectively. The data demonstrate no correlation between these two parameters in F(M197)R RC; however, it might be worth considering the possible integration of this new water molecule into the hydrogen-bonding network, which could affect the redox potential of P, as discussed below.

The structure of F(M197)H RC does not provide a straightforward explanation why the opposite mutations F(M197)H and H(L168)F bring about different changes in the *E*_m_ P/P^+^ (Lin *et al.*, 1994 ▸; Mattioli *et al.*, 1994 ▸). However, it implies that there are some additional mechanisms for fine-tuning the P redox potential and indicates that these mechanisms may be associated with the sizes and/or the patterns of the hydrogen-bonding networks involving the L168 and M197 residues. There are experimental data in support of this assumption showing that the redox properties of the primary electron donor can be regulated by modification of the hydrogen-bonding network involving His L168 near BChl P_A_. It has been demonstrated that the substitution of Asn L166 by His or Asp in the *R. sphaeroides* RC resulted in the strengthening of the hydrogen bond of His L168 to the acetyl carbonyl group of P_A_, which was presumably associated with strengthening of the hydrogen bonding of the L166 residue to His L168. These mutations were accompanied by a notable increase in the P/P^+^ midpoint potential (Ivancich *et al.*, 1997 ▸).

An alignment of the protein sequences of RC subunits showed that those species of purple bacteria that had RC-associated tetraheme cytochromes (four-subunit RCs) possessed conserved His residues at the L166 position. Therefore, following the above, it may be expected that these RCs have a stronger hydrogen bond connecting BChl P_A_ and His L168, as well as a higher P redox potential compared with that in the three-subunit RCs of *R. sphaeroides*, *R. capsulatus* and *Rhodospirillum rubrum*, in which the L166 site is occupied by Asn. In contrast to the predictions, the value of the *E*_m_ P/P^+^ in the *Rhodospirillum centenum* four-subunit RC was found to be 25 mV lower than that of *R. sphaeroides* (Wang *et al.*, 1994 ▸). This is despite the fact that, as in other known four-subunit RCs, the acetyl group of BChl P_B_ is hydrogen-bonded to the conserved Tyr M195, which should also increase the redox potential of the primary electron donor. It was assumed that the presence of the two hydrogen bonds that the P dimer forms with its environment, as well as the tuning of the redox potential of P by changing the strength of the interaction between the L166 residue and His L168, affecting the strength of the His L168–P_A_ hydrogen bond, can serve to optimize the redox compatibilities between the RC-associated cytochrome, P and the primary quinone acceptor (Ivancich *et al.*, 1997 ▸; Ullmann *et al.*, 2008 ▸).

**Figure 7:**
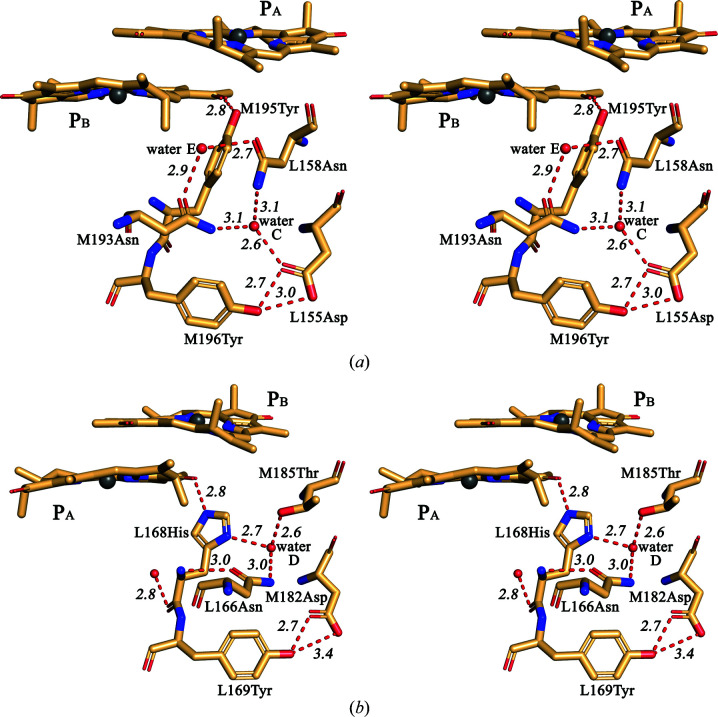
Stereoviews of the hydrogen-bonding networks near Tyr M195 (*a*) and His L168 (*b*) in *B. viridis* RC. All distances are shown in Å.

Two high-resolution structures are available of four-subunit RCs from purple bacteria. In *Blastochloris viridis* RC three amino acids donate hydrogen bonds to BChls P_A_ and P_B_ (Lancaster & Michel, 1997 ▸): Tyr at position M195 (the analog of the M197 position in *R. sphaeroides*), Thr at position L248 and His at position L168. Tyr M195 is hydrogen-bonded to the acetyl carbonyl group of P_B_ and is not connected to the existing hydrogen-bonding network [PDB entry 2i5n; Li *et al.*, 2006 ▸; Fig. 7 ▸(*a*)]. Besides, the L166 position in *B. viridis* RC is occupied by Asn [Fig. 7 ▸(*b*)] and not by His as in other known four-subunit RC complexes, which, as mentioned above, resulted in a weakening of the hydrogen bond between P_A_ and His L168 in *R. sphaeroides* RC. However, the pattern of the hydrogen-bonding network involving His L168 in *B. viridis* RC appears to be more similar to the pattern in *R. sphaeroides* RC on the P_B_ side near His M197, with a symmetry-related analog of water E present there [Figs. 7 ▸(*b*) and 4 ▸(*b*)]. The only noticeable difference is that water D appears to be hydrogen-bonded not to Asp M182 but to Thr M185. We assume that despite three keto groups of P in *B. viridis* RC being hydrogen-bonded to their protein environment, which should notably increase the redox potential of the primary electron donor, the modification of the hydrogen-bond network near His L168 could contribute to fine-tuning of the P potential, leading to an interesting fact: the value of the *E*_m_ P/P^+^ in this RC is only 20 mV higher than that in *R. sphaeroides*, namely 520 mV (Huppman *et al.*, 2002 ▸). The cumulative effect of the two substitutions, Y(M195)H and Y(M195)F, on the P/P^+^ midpoint potential in *B. viridis* RC was also surprising, with values of +20 and −45 mV, respectively (Huppman *et al.*, 2002 ▸), which are twice as low compared with the effect of the F(M197)H mutation in the *R. sphaeroides* RC (+125 mV). A possible explanation of this fact follows from Fig. 7 ▸(*a*). In *B. viridis* Y(M195)H RC His M195 cannot be connected to the hydrogen-bonding network through water C, since this water already forms three hydrogen bonds to the surrounding residues. In this regard, why the Phe→Tyr M197 substitution in *R. sphaeroides* RC, which does not lead to the incorporation of BChl P_B_ into the hydrogen-bonding network, only increases the oxidation potential of P by 30 mV (Wachtveitl *et al.*, 1993 ▸), while the F(M197)H mutation connecting the P_B_ molecule to this network causes a much greater increase in the *E*_m_ P/P^+^ (Lin *et al.*, 1994 ▸), can also be explained. It should be noted that the strengths of the individual hydrogen bonds formed by Tyr and His M197 with the P_B_ acetyl group are nearly the same at 3 and 3.5 kcal mol^−1^, respectively (Ridge *et al.*, 2000 ▸). The high-resolution structure of the RC from the purple sulfur bacterium *Thermochromatium tepidum* shows another interesting case of hydrogen-bonding networks near P_A_ (Yu *et al.*, 2018 ▸; PDB entry 5y5s). There is no water D in the structure, and the conserved residues His, Tyr and Asp, which are analogous to Asn L166, Tyr L169 and Asp M182, respectively [Fig. 7 ▸(*b*)], are located so close that they can form hydrogen bonds directly through their side groups. Both waters C and E are present at the P_B_ side, but the pattern of the hydrogen-bonding network is not exactly the same as in *B. viridis* RC (Yu *et al.*, 2018 ▸; PDB entry 5y5s). Taken together, these data indicate that in the RCs from purple bacteria the hydrogen-bonding networks in the vicinity of dimer P appear to participate in fine-tuning the redox properties of the primary electron donor. As mentioned above, Figs. 4 ▸(*b*) and 6 ▸ show differences in the two hydrogen-bonding networks near the M197 and L168 sites. In the structure of *R. sphaeroides* F(M197)H RC, His M197 directly interacts with water C, while in the WT RC His L168 contacts Asn L166. Asn M195, the symmetry-related residue to Asn L166, is hydrogen-bonded to Ser L158 through water E, and this part of the network is absent on the L168 side. The other molecules that participate in the hydrogen-bonding interactions on both sides are similar. We assume that these dissimilarities may account for the different changes of the *E*_m_ P/P^+^ in the F(M197)H RC and H(L168)F RC.

Regulation of the potentials of redox proteins by changing the total strength of hydrogen bonds in the networks around the redox-active center is known for other participants in electron-transport pathways (Lin *et al.*, 2005 ▸). For example, in the iron–sulfur protein rubredoxin from *Clostridium pasteurianum* the redox-active center consists of an Fe atom coordinated through the S atoms of four cysteine residues. In addition, the S atoms of these cysteines form six hydrogen bonds to the amide side groups of the surrounding residues. It has been shown that the 126 mV span of reduction potentials of ten rubredoxin mutant variants is associated with changes in the strengths of hydrogen bonds resulting from the mutations. The authors emphasize that aggregate changes in multiple hydrogen bonds, rather than changes in a single hydrogen bond, must be taken into consideration in order to explain the changes of the reduction potential in this electron-transport protein (Lin *et al.*, 2005 ▸).

### 3.5. Structural basis for the increased thermal stability of F(M197)H RC

Previous analysis of the *R. sphaeroides* RC structure revealed two clefts at the interface of the L and M subunits: one near the M197 position and another one near the symmetry-related L168 position (McAuley-Hecht *et al.*, 1998 ▸). The results of our work demonstrate that in the WT RC the residues of the L and M subunits lining the opposite sides of these clefts are hydrogen-bonded. Three such interactions are seen near the M197 position, coupling Asp L155 with Tyr M198, Asp L155 with Asn M195 via water C, and Ser L158 with Asn M195 via water E [Fig. 4 ▸(*a*)]. The L and M subunits on the L168 side seem to be linked through Asp M184, which can form two hydrogen bonds: one to Tyr L169 and another to Asn L166 via water D (Fig. 6 ▸). It can be assumed that overall these hydrogen bonds contribute to the stability of the RC, preventing the hydrophobic core of the membrane protein from being exposed to the aqueous phase (Haltia & Freire, 1995 ▸). It is not surprising that the A branch that is active in photosynthetic electron transfer appears to be better protected in this respect.

The increased thermal stability of F(M197)H RC, as well as its stability in response to external pressure, were rationalized in terms of the new hydrogen bonds that are formed in this complex (Holden-Dye *et al.*, 2011 ▸; Kangur *et al.*, 2017 ▸). It was noted that the opposing changes of stability seen in the F(M197)H and H(L168)F mutants might not be a simple consequence of the introduction or removal of a protein–cofactor hydrogen bond *per se*, but rather be specific to the Phe→His exchange at these particular positions in the M or L polypeptide (Holden-Dye *et al.*, 2011 ▸). The structural data obtained in this work are in agreement with this assumption. It seems likely that not just the formation of two new hydrogen bonds but also the involvement of His M197 in the existing hydrogen-bonding network is related to the strengthening of the inter-protein interactions near the periplasmic surface of the membrane and may be responsible for the stabilization of the F(M197)H RC structure. It has been reported that dynamic hydrogen-bonding networks can provide the structural plasticity required for the functioning of some other soluble and membrane bacterial proteins (Karathanou & Bondar, 2018 ▸). The participation of His L168 in analogous through-bond contacts appears to account for the related decrease in stability of H(L168)F RC. It has previously been shown that the rate of the initial electron transfer in H(L168)F RC was faster than that in the WT RC (Lancaster *et al.*, 2000 ▸; Wang *et al.*, 2012 ▸). Although it is known that the natural design of photosynthetic RCs is maximally optimized and the quantum yield of charge separation in RC is extremely high, close to 100%, we can put forward a proposal why this mutation did not take root in the process of evolution of the RCs. The rise in the photosynthetic electron transfer rate by the H(L168)F substitution would be achieved at the cost of decreased stability of the RC complex. In addition, it was noted that a blue shift of the Q_*y*_ P absorption band in H(L168)F mutant RCs makes energy transfer from light-harvesting antennae less efficient (Lancaster *et al.*, 2000 ▸), and the increased charge recombination process does not contribute to a high yield in the photochemical reaction (Wang *et al.*, 2012 ▸). Given the proven formation of one hydrogen bond to the P dimer that is observed in the L(M160)H and L(L131)H RCs, the small or even opposite effect of these mutations on the stability of the RC could be related to the details of their specific location and/or to the absence of additional interactions of the imidazoles with their surroundings that are needed to strengthen the protein structure (Holden-Dye *et al.*, 2011 ▸). However, the absence of high-resolution crystal structures of these mutant complexes does not allow clear conclusions to be made in this respect.

## 4. Conclusions

Analysis of the crystal structure of the *R. sphaeroides* RC with a Phe→His substitution at the M197 position revealed that the formation of a hydrogen bond between His M197 and the acetyl carbonyl group of P_B_ resulted in an ∼33° out-of-plane rotation of this group. In addition, the distance between BChl P_A_ and P_B_ in the area of pyrrole ring I was found to be altered by ∼0.10–0.17 Å. These structural changes, as predicted by computational studies, have the opposite effect on the energy of the Q_*y*_ P transition, which explains the unchanged position of the Q_*y*_ P band in the absorption spectrum of F(M197)H RC. It was shown that as a result of the F(M197)H mutation, the imidazole group of His M197 became a link connecting BChl P_B_ to an extensive hydrogen-bonding network in its protein environment. Comparison of the patterns of the networks involving histidines L168 and M197 on the two sides of the P dimer shows certain differences that may be responsible for fine-tuning the redox properties of the primary electron donor. The structure of F(M197)H RC provides details that explain the increased stability of this complex.

## Supplementary Material

PDB reference: F(M197)H mutant structure of photosynthetic reaction center, 7od5

PDB reference: 7p2c

## Figures and Tables

**Table 1:** Data collection and processing for F(M197)H RC Values in parentheses are for the highest resolution shell.

	One crystal, 100 K	Multi-crystal, RT
Diffraction source	Beamline P11, PETRA III, DESY	Beamline P11, PETRA III, DESY
Wavelength (Å)	1.0332	0.4769
Temperature (K)	100	293
Detector	PILATUS 6M-F	PILATUS3 X 2MCdTe
Crystal-to-detector distance (mm)	390.8	400
Rotation range per image (°)	0.1	0.1
Total rotation range (°)	180	
Exposure time per image (s)	0.05	0.1
Space group	*P*4_2_2_1_2	*P*4_2_2_1_2
*a*, *b*, *c* (Å)	99.98, 99.98, 238.12	102.5, 102.5, 237.4
Mosaicity (°)	0.084	0.14–0.61
Resolution range (Å)	50–2.10 (2.15–2.10)	47.05–2.04 (2.08–2.04)
Total No. of reflections	941920 (152790)	2329422 (129121)
No. of unique reflections	71237 (11313)	81278 (4373)
Completeness (%)	99.9 (99.8)	100.0 (99.9)
Multiplicity	13.22 (13.50)	28.7 (29.5)
〈*I*/σ(*I*)〉	16.93 (1.79)	10.2 (0.7)
*R*_r.i.m._	0.120 (1.362)	0.057 (1.025)
CC_1/2_	0.99 (0.69)	0.998 (0.403)

**Table 2:** Structure solution and refinement for F(M197)H RC Values in parentheses are for the highest resolution shell.

	One crystal, 100 K	Multi-crystal, RT
PDB code	7od5	7p2c
Resolution range (Å)	46.07–2.10 (2.15–2.10)	47.05–2.04 (2.06–2.04)
Completeness (%)	99.95 (99.0)	99.92 (100.0)
σ Cutoff [*F* > σ(*F*)]	1.36	1.34
No. of reflections, working set	71233 (4524)	81233 (2610)
No. of reflections, test set	2101 (138)	4079 (133)
Final *R*_cryst_ (%)	18.05 (25.05)	20.24 (31.02)
Final *R*_free_ (%)	22.07 (31.56)	22.90 (33.15)
R.m.s. deviations		
Bond lengths (Å)	0.007	0.007
Angles (°)	0.903	0.929
Average *B* factors (Å^2^)		
Protein	40.0	41.57
Ligand	44.9	47.65
Water	40.5	38.86
Calculated DPI (Å)	0.156	0.149
Maximal estimated error (Å)	0.096	0.093
Ramachandran plot		
Most favored (%)	97.92	97.43
Allowed (%)	2.08	2.57

**Table 3:** Distances between BChl P_A_ and P_B_ in the area of pyrrole ring I (Å)

	WT RC, one crystal, 100 K (PDB entry 6z27)	F(M197)H RC, one crystal, 100 K (PDB entry 7od5)	WT RC, multi-crystal, RT	F(M197)H RC, multi-crystal, RT (PDB entry 7p2c)
P_A_ MG–CBB P_B_	3.84	3.52 (0.32)†	3.76 (0.08)†	3.43 (0.33)‡
P_A_ NB–C3B P_B_	3.43	3.53 (0.10)	3.49 (0.06)	3.66 (0.17)
P_A_ C1B–C2B P_B_	3.46	3.57 (0.11)	3.55 (0.09)	3.72 (0.17)
P_A_ C2B–C1B P_B_	3.53	3.50 (0.03)	3.58 (0.05)	3.64 (0.06)
P_A_ C3B–NB P_B_	3.58	3.47 (0.11)	3.54 (0.04)	3.60 (0.06)
P_A_ C4B–C4B P_B_	3.51	3.49 (0.02)	3.49 (0.02)	3.57 (0.08)
P_A_ CBB–MG P_B_	3.49	3.73 (0.24)	3.48 (0.01)	3.87 (0.39)

† The deviation from the wild-type structure at 100 K.

‡ The deviation from the wild-type structure at RT.
